# Efficacy-mediated effects of spirituality and physical activity on quality of life: A path analysis

**DOI:** 10.1186/1477-7525-10-57

**Published:** 2012-05-29

**Authors:** James F Konopack, Edward McAuley

**Affiliations:** 1 Department of Kinesiology and Community Health, University of Illinois at Urbana-Champaign, Urbana, IL, USA; 2 Department of Nursing and Health Studies, Monmouth University, West Long Branch, NJ, USA

**Keywords:** Self-efficacy, Quality of life, Physical activity, Spirituality

## Abstract

**Background:**

Physical activity has been established as an important determinant of quality of life, particularly among older adults. Previous research has suggested that physical activity’s influence on quality of life perceptions is mediated by changes in self-efficacy and health status. In the same vein, spirituality may be a salient quality of life determinant for many individuals.

**Methods:**

In the current study, we used path analysis to test a model in which physical activity, spirituality, and social support were hypothesized to influence global quality of life in paths mediated by self-efficacy and health status. Cross-sectional data were collected from a sample of 215 adults (male, *n* = 51; female, *n* = 164) over the age of 50 (*M* age = 66.55 years).

**Results:**

The analysis resulted in a model that provided acceptable fit to the data (*χ*2 = 33.10, df = 16, p < .01; RMSEA = .07; SRMR = .05; CFI = .94).

**Conclusions:**

These results support previous findings of an efficacy-mediated relationship between physical activity and quality of life, with the exception that self-efficacy in the current study was moderately associated with physical health status (.38) but not mental health status. Our results further suggest that spirituality may influence health and well-being via a similar, efficacy-mediated path, with strongest effects on mental health status. These results suggest that those who are more spiritual and physically active report greater quality of life, and the effects of these factors on quality of life may be partially mediated by perceptions of self-efficacy.

## Background

Self-reported quality of life has been positively associated with measures of spirituality, such as a perceived connection with the divine [1] and private religious practice [2]. It has been suggested that spirituality may confer quality of life benefits independent of other factors [3], but most published work has focused on spirituality’s connection with specific health outcomes rather than with global measures of quality of life. Indeed, the literature is replete with studies linking spirituality to various health outcomes. For example, it has been reported that religious individuals have a lower risk for morbidity and mortality [4,5] and tend to perceive themselves with less disability than do less religious individuals [6]. However, despite these findings and a growing attention to spiritual matters in healthcare, relatively little has been published on likely explanatory mechanisms underlying such relationships.

Self-efficacy is a construct that has been suggested as a mediator of the relationship between spirituality and well-being. It has been speculated that spirituality may help some individuals to “gain a sense of control over their lives” [7]. The possibility of mediation by self-efficacy or control constructs in general has long been supported, even if implicitly, in the literature [1,8-10] and echoes the ideas of spiritual modeling and “partnered proxy agency” suggested by Bandura [11], yet empirical investigation of this hypothetical association is lacking. Efficacy-mediated models have been empirically tested and validated in another context, however.

Research published by McAuley, Konopack, Motl, Morris, Doerksen, and Rosengren [12] demonstrated support for a model in which self-efficacy mediated physical activity’s effects on quality of life. In their study, McAuley et al. [12] operationalized mental and physical health status as proximal indicators of global quality of life. They found that the direct relationship between physical activity and health status was rendered non-significant when self-efficacy was introduced into the model, thereby demonstrating mediation by self-efficacy. Subsequent research has found support for a similar efficacy-mediated model of the relationship between physical activity and quality of life [13]. Thus, evidence exists to support self-efficacy as a reliable mediator of physical activity’s influence on quality of life.

When examining the relationship between spirituality and quality of life, others have positioned health status as a mediating variable [14]. Although the authors cited others’ work with factors such as health behaviors and self-care agency in the context of their discussion of the spirituality-quality of life relationship, that study did not include specific measurement of these constructs. Thus, there is theoretical support in the literature for self-efficacy as a mediator of both physical activity’s and spirituality’s effects on quality of life, but this relationship has yet to be explicitly tested.

To address this question in the present study, we attempted to replicate the model of the physical activity and quality of life relationship first published by McAuley and colleagues [12], expanded here to examine self-efficacy as a mediator of the association between spirituality and quality of life. For both physical activity and spirituality, the influences on quality of life were hypothesized to operate through both self-efficacy and physical and mental health status.

## Methods

### Participants

Adults ages 50 years and above were recruited from the local community through electronic mail, newspaper advertisements, snowball sampling via previous research participants, and announcements made and flyers distributed in local religious and community centers. Individuals volunteering to participate were deemed eligible if they were willing and able to complete paper-and-pencil questionnaires and wear an accelerometer for one week, were 50 years of age or older at time of contact, and were able to pass a basic cognitive screening [15] to ensure validity of questionnaire responses. A total sample of 215 individuals provided data. Participants were primarily female (*n* = 164, 76.3%) and White/Caucasian (*n* = 191, 88.8%) and ranged in age from 50–84 years (*M* age = 66.55 years ± 9.44). Demographic data from the study sample are presented in Table 1.

**Table 1:** Demographic data from the study sample

	***M (SD)* or category**	**Frequency**	**Percentage**
Age	66.55 (9.44)	-	-
Pfeiffer score	7.64 (0.55)	-	-
Race	White	191	88.8
	Black	16	7.4
	Asian	4	1.9
	Other/Multi-racial	4	1.9
Ethnicity	Hispanic/Latino	2	0.9
	Non-Hispanic/-Latino	213	99.1

### Measures

After signing an institutionally-approved informed consent form, participants completed the following measures:

#### Quality of life

Quality of life was assessed using the Satisfaction with Life Scale (SWLS) [16], a 5-item scale developed to assess global life satisfaction across various age groups. Each scale item is rated on a 7-point scale from strongly disagree (1) to strongly agree (7), with higher scores representing greater life satisfaction. This instrument has been used as a quality of life measure in a number of investigations involving physical activity and older adults [12,17].

#### Health status

The 12-Item Short Form Survey (SF-12) [18], a shortened version of the Medical Outcomes Study SF-36 Health Survey [19], was developed out of a need for brevity in large-scale health studies that could not be met with the larger SF-36. In the current study, the Mental Health and Physical Health summary scores were used as measures of mental and physical health status, respectively.

#### Social support

Social support was measured using an abbreviated version the Social Provisions Scale [20], which assesses 6 different social provisions in accordance with previous work on the subject by Weiss [21]: *attachment* (i.e., emotional support), *social integration* (i.e., existing social network), *reassurance of worthreliable alliance* (i.e., tangible aid), *guidance*, and *opportunity for nurturance*.

#### Self-efficacy

The Lifestyle Physical Activity Self-Efficacy Scale (LSE) [22] was designed to assess confidence in one’s ability to accumulate 30 minutes of physical activity on 5 or more days of the week for incremental one-month periods, from one month to six months. In the present study, the LSE was used as a measure of self-efficacy specific to physical activity.

The Self-Care Self-Efficacy Scale (SCSE) [23] assesses an individual’s confidence in his or her ability to cope with self-care challenges due to a situation such as illness. With the permission of the developer (Dr. Lev), the scale was modified for use in the current study by replacing language specific to illness with language referring to the aging process in general. The original measure has demonstrated evidence of validity in previous studies [23].

#### Physical activity

Physical activity data were collected using the Actigraph portable accelerometer (Actigraph, LLC, Pensacola, FL). The Actigraph accelerometer has been shown to provide valid assessments of physical activity level in adult men and women during treadmill walking, running and daily activity [24,25]. Previous work has demonstrated that the Actigraph accelerometer accurately predicts energy expenditure and demonstrates superior reliability when compared with other accelerometers [26,27]. Actigraph data in the present study are reported as the total number of activity counts per day, averaged across a three-day period.

#### Spirituality and religiousness

Measurement of spirituality and religiousness in the current study was accomplished using two items selected from the Overall Self-Ranking dimension of the Brief Multidimensional Measure of Religiousness/Spirituality (BMMRS) [28], an instrument that showed evidence of reliability and validity when psychometrically evaluated in the 1998 General Social Survey [29]. In the current investigation, participants indicated the extent to which they considered themselves “spiritual” or “religious” by selecting a response along a 4-point Likert-type scale for each of the following questions: “To what extent do you consider yourself a religious person?” and “To what extent do you consider yourself a spiritual person?”

### Data analysis

A model in which spirituality, social support, and physical activity influenced hierarchical quality of life in a parallel fashion was specified in a path analysis using M*plus* version 3.21 covariance modeling software [30]. Model-to-data fit in the current study was evaluated using the chi-square test [31] and root mean square error of approximation (RMSEA) [32] statistics in combination with the comparative fit index (CFI) and standardized root mean square residual (SRMR), which are accepted indicators of model-data fit [30,33]. The strength of relationships between study variables was estimated using standardized path coefficients.

## Results

### Model-to-data fit

**Figure 1:**
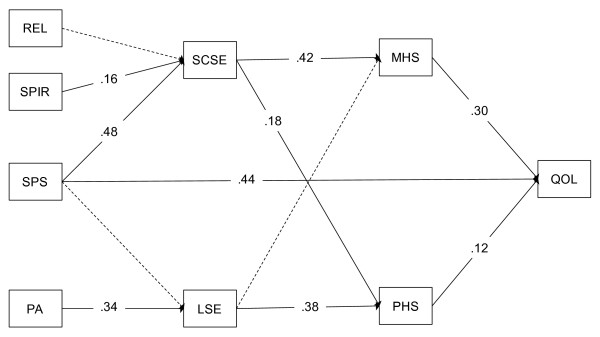
**Model results depicting observed paths among study variables.** REL = religiousness, SPIR = spirituality, SPS = social provisions, PA = physical activity, SCSE = self-care self-efficacy, LSE = lifestyle physical activity self-efficacy, MHS = mental health status, PHS = physical health status, QOL = quality of life.

The hypothetical model provided a good fit to the data according to traditional structural equation modeling fit indices (*χ*2 = 33.10, df = 16, p < .01; RMSEA = .07; SRMR = .05; CFI = .94). Significance in the chi-square statistic, which is generally indicative of a poor-fitting model, is typically tolerated in evaluating the fit of hypothesized models in data sets containing a large number of observations [34]. The hypothetical model tested in this study, which can be seen with standardized path coefficients in Figure 1, accounted for significant variance in quality of life scores (*R*^2^ = .35).

### Mediation by self-efficacy

Physical activity, social support, and spirituality each accounted for significant variance in associated self-efficacy constructs, with standardized path coefficients (βs) of .34, .48, and .16, respectively. These efficacy constructs, in turn, accounted for significant variance in mental and physical health status, confirming initial study hypotheses. These results were similar to those observed by McAuley and colleagues [12], with the exception that physical activity self-efficacy was moderately associated with physical health status (β = .38) but not mental health status (β = .10).

The efficacy-mediated influence of spirituality was observed to be stronger for mental health status (β = .42) than for physical health status (β = .18). Thus, more spiritual individuals reported greater self-care self-efficacy, which, in turn, was associated with more positive health status. This association was stronger with mental health status than with physical health status. In addition to its effects on self-efficacy, social support was observed to maintain a statistically significant direct relationship with global quality of life (standardized path coefficient = β = 44), indicating the quality of life benefits derived from social provisions, above and beyond the effects of physical activity and spirituality.

## Discussion

The results of this study provide further support for previously proposed efficacy-mediated models of physical activity and quality of life [12,13]. More importantly, the results reported here provide initial evidence for the extension of McAuley et al.’s [12] hierarchical, social cognitive model to understanding the association between spirituality and quality of life. Specifically, our data suggest that spirituality may exert an influence on health and well-being in a path that, like physical activity, is mediated by self-efficacy.

In our best-fitting model, spirituality exhibited a stronger connection with mental health status than with physical health status. These results are similar to the findings of Sawatzky and colleagues [14], who, in their study of spirituality among adolescents, also found mental health status to mediate the association between spirituality and quality of life. Our data suggest that spirituality’s influence on quality of life operates largely through mental health status, and physical activity’s influence on quality of life is chiefly through physical health status. Although previous research has certainly supported physical activity as a mental health determinant, there is also evidence for spirituality as a determinant of physical health above and beyond the influence of psychosocial factors [35]. Indeed, recent evidence supports our findings that physical activity’s effects on physical health status are stronger than on mental health status, and that global quality of life is more strongly influenced by mental health status [36]. Thus, our results suggest that physical activity and spirituality are complementary determinants of quality of life, with their strongest influences on physical and mental health status, respectively.

We also observed a direct path between the provision of social support and perceptions of global quality of life that was significant above and beyond the effects on self-efficacy (β = .44), as shown in Figure 1. Others have similarly found social support to be an important variable to consider when examining the extent to which spirituality influences health outcomes. For example, an investigation of quality of life among Korean and Korean American breast cancer survivors resulted in social support for the mediating influence of spirituality, but only for Korean Americans [37]. At present, it appears that some of the quality of life benefits derived from spirituality are due to increases in social support, yet the manner in which social support operates in a hierarchical model of quality of life may differ across populations. Certainly, social support remains an important determinant of quality of life [38], and future research in this area is warranted.

Programs and services designed to improve quality of life among older adults are needed as the population in the United States continues to face increasing age-related challenges to health and functioning. Targeting a modifiable construct like self-efficacy may help, in this respect [39]. The results of the current study provide additional support for the mediating role of self-efficacy perceptions in the determination of health status and global quality of life. Our data tentatively suggest that programs designed to promote physical activity and feelings of spirituality – but not necessarily religiousness – will likely have a greater impact if they also target self-efficacy.

The current study was not without its limitations, one of which was the small number of racial minorities that took part in the study. Despite concerted efforts to recruit an ethnically diverse sample over the course of the study, small numbers of minorities participated. Minority participants lower scores on lifestyle self-efficacy, social provisions, and satisfaction with life, as can be seen in Table 2. An insufficient number of minorities precluded our examination of whether racial status influenced the strength of the paths in our model, so further work is needed to examine might possibly moderate these relationships. Some diversity was observed with respect to the religious affiliation of participants, with 21 individuals (9.8% of the sample) reporting affiliation with a religion outside of Judeo-Christianity (e.g., Buddhism, Hinduism, others), 8 participants (3.7%) identifying themselves as atheist or “none,” and another 8 participants (3.7%) identifying themselves as Jewish. Still, 31 individuals (14.4%) identified themselves as Catholic, and 147 (68.4%) reported affiliation with other Christian denominations. Thus, future research is needed to ascertain whether the relationships among variables reported in the current study among older adults exist among even populations with greater diversity with respect to age and religious affiliation.

**Table 2:** Mean, standard deviation, and range of observed variables

**Variable**	**Total Sample**	**Range**	**White (*n* = 194)**	**Minority (*n* = 24)**
Satisfaction with Life	26.42 (5.82)	10-35	26.75 (5.67)*	23.83 (6.47)
Social Provisions	20.80 (2.77)	12-24	20.93 (2.67)*	19.75 (3.40)
Self-Care Self-Efficacy	64.22 (9.96)	21-80	64.41 (9.51)	62.69 (13.14)
Lifestyle Self-Efficacy	73.83 (31.79)	0-100	75.73 (30.75)*	59.68 (36.33)
Religiousness	2.99 (0.86)	1-4	2.94 (0.88)*	3.38 (0.65)
Spirituality	3.26 (0.77)	1-4	3.23 (0.79)	3.50 (0.59)
Physical Health	48.78 (9.18)	15.97-63.83	49.13 (8.75)	45.96 (11.91)
Mental Health	53.58 (7.43)	27.32-67.73	53.75 (7.29)	52.16 (8.54)
Average accelerometer counts per day	236294 (109142)	56646-555000	236294 (109142)	206209 (91679)

* Statistically significant (*p* < .05) difference between racial categories

Although the path analysis reported here corroborated and extended existing research, the data were cross-sectional, thereby limiting the extent of our ability to draw causal inferences. One final question that remains is that of which efficacy measure to use. It is clear from the results in this study that religiosity was not related to self-care self-efficacy. Yet, in the religiosity literature, control constructs are repeatedly suggested to mediate the beneficial aspects of religiosity on health and well-being outcomes. If, as Bandura [11] suggested, this can be explained by “partnered proxy efficacy,” the question becomes: Self-efficacy with respect to what, if not self-care? The challenge remains to precisely determine which control constructs are driving the effects of spirituality on well-being.

## Conclusions

The data presented here provide support for a hypothetical model in which self-efficacy mediates the relationship between physical activity and quality of life. Moreover, evidence was also provided for a similarly structured, efficacy-mediated path between spirituality and quality of life. Thus, it appears that control constructs such as self-efficacy account for some portion of the quality of life benefits derived from both spirituality and physical activity. Further investigation of these relationships, particularly the influence of spirituality on health and quality of life outcomes, is needed.

## Competing interests

The authors declare that they have no competing interests.

## Authors’ contributions

JFK contributed to the design of the study, carried out the recruitment of participants, conducted principal data analyses, and drafted the original and revised manuscript. EM contributed to the design of the study, assisted with data analyses, and helped with the drafting of the manuscript. All authors read and approved the final manuscript.

## References

[B1] Pollner M Divine relations, social relations, and well-being J Heal Soc Behav 1989 30 92 104 10.2307/2136915 2470806

[B2] Diener E Clifton D Life satisfaction and religiosity in broad probability samples Psychol Inq 2002 13 206 209

[B3] Pargament KI Is religion nothing but …? Explaining religion versus explaining religion away Psychol Inq 2002 13 239 244 10.1207/S15327965PLI1303_06

[B4] Hill TD Angel JL Ellison CG Angel RJ Religious attendance and mortality: An 8-year follow-up of older Mexican Americans Journals of Gerontology B: Psychological Sciences and Social Sciences 2005 60 S102 S109 10.1093/geronb/60.2.S102 15746025

[B5] Koenig HG McCullough M Larson D Handbook of religion and health 2001 Oxford University Press, Oxford

[B6] Idler EL Religious involvement and the health of the elderly: Some hypotheses and an initial test Social Forces 1987 66 226 238

[B7] Siegel K Schrimshaw EW The perceived benefits of religious and spiritual coping among older adults living with HIV/AIDS Journal for the Scientific Study of Religion 2002 41 91 102 10.1111/1468-5906.00103

[B8] Levin JS Religion and health: is there an association, is it valid, and is it causal? Social Science & Medicine 1994 38 1475 1482 10.1016/0277-9536(94)90109-0 8036527

[B9] Mattis JS Jagers RJ A relational framework for the study of religiosity and spirituality in the lives of African Americans Journal of Community Psychology 2001 29 519 539

[B10] Strawbridge WJ Shema SJ Cohen RD Kaplan GA Religious attendance increases survival by improving and maintaining good health behaviors, mental health, and social relationships Annals of Behavioral Medicine 2001 23 68 74 10.1207/S15324796ABM2301_10 11302358

[B11] Bandura A On the psychosocial impact and mechanisms of spiritual modeling Int J Psychol Relig 2003 13 167 173 10.1207/S15327582IJPR1303_02

[B12] McAuley E Konopack JF Motl RW Morris KS Doerksen SE Rosengren K Physical activity and quality of life in older adults: Influence of health status and self-efficacy Annals of Behavioral Medicine 2006 31 99 103 10.1207/s15324796abm3101_14 16472044

[B13] White SM Wójcicki TR McAuley E Physical activity and quality of life in community dwelling older adults Health and Quality of Life Outcomes 2009 7 10 10.1186/1477-7525-7-10 19200385 PMC2649048

[B14] Sawatzky R Gadermann A Pescut B An investigation of the relationships between spirituality, health status and quality of life in adolescents Applied Research in Quality of Life 2009 4 5 22 10.1007/s11482-009-9065-y

[B15] Pfeiffer E A short portable mental status questionnaire for the assessment of organic brain deficit in elderly patients J Am Geriatr Soc 1975 23 433 441 1159263 10.1111/j.1532-5415.1975.tb00927.x

[B16] Diener E Emmons RA Larsen RJ Griffin S The Satisfaction with Life Scale J Personal Assess 1985 49 71 75 10.1207/s15327752jpa4901_13 16367493

[B17] Elavsky S McAuley E Motl RW Konopack JF Marquez DX Jerome GJ Physical activity enhances long-term quality of life in older adults: Efficacy, esteem, and affective influences Annals of Behavioral Medicine 2005 30 138 145 10.1207/s15324796abm3002_6 16173910

[B18] Ware JE Kosinski M Keller SD The SF-36 Physical and Mental Health Summary Scales: A user's manual. Boston, MA 1994 The Health Institute, New England Medical Center

[B19] Ware JE The status of health assessment 1994 Annual Review of Public Health 1995 16 327 354 10.1146/annurev.pu.16.050195.001551 7639876

[B20] Russell DR Cutrona C Jones WH, Perlman D The provisions of social relationships and adaptation to stress In Advances in personal relationships 1984 JAI Press, Greenwich, CT 37 68

[B21] Weiss RS The provisions of social relationships 1974 Prentice Hall, Englewood Cliffs, NJ

[B22] McAuley E Jerome GJ Marquez DX Elavsky S Blissmer B Exercise self-efficacy in older adults: Social, affective, and behavioral influences Annals of Behavioral Medicine 2003 25 1 7 10.1207/S15324796ABM2501_01 12581930

[B23] Lev EL Owen SV A measure of self-care self-efficacy Res Nurs Heal 1996 19 421 429 10.1002/(SICI)1098-240X(199610)19:5<421::AID-NUR6>3.0.CO;2-S 8848626

[B24] Melanson EL Freedson PS Validity of the Computer Science and Applications, Inc: CSA) activity monitor Medicine & Science in Sports & Exercise 1995 27 934 940 7658958

[B25] Sirard JR Melanson EL Li L Freedson PS Field evaluation of the Computer Science and Applications, Inc. physical activity monitor Medicine & Science in Sports & Exercise 2000 32 695 700 10731015 10.1097/00005768-200003000-00022

[B26] Welk GJ Blair SN Wood K Jones S Thompson RW A comparative evaluation of three accelerometry-based physical activity monitors Medicine & Science in Sports & Exercise 2000 32 Suppl 9 489 497 10.1097/00005768-200009001-00008 10993419

[B27] Welk GJ Schaben JA Morrow JRJ Reliability of accelerometry-based activity monitors: A generalizability study Medicine & Science in Sports & Exercise 2004 36 1637 1645 15354049

[B28] National Institute on Aging/Fetzer Workgroup Multidimensional measurement of religiousness/spirituality for use in health research 1999 John E. Fetzer Institute, Kalamazoo, MI

[B29] Idler EL Musick MA Ellison CG George LK Krause N Ory MG Measuring multiple dimensions of religion and spirituality for health research: Conceptual background and findings from the 1998 General Social Survey Research on Aging 2003 25 327 365 10.1177/0164027503025004001

[B30] Muthén LK Muthén BO Mplus 1998 321 Muthén & Muthén, Los Angeles

[B31] Bollen KA Structural equations with latent variables 1989 Wiley-Interscience, New York

[B32] Browne MW Cudeck R Bollen KA, Long JS Alternative ways of assessing model fit In Testing structural equation models 1993 Sage Publications, Newbury Park, CA 136 162

[B33] Hu L Bentler PM Fit indices in covariance structure modeling: sensitivity to underparameterized model misspecification Psychological Methods 1998 3 424 453

[B34] Bentler PM Bonett DG Significance tests and goodness of fit in the analysis of covariance structures Psychol Bull 1980 88 588 606

[B35] Lawler-Row KA Elliott J The role of religious activity and spirituality in the health and well-being of older adults J Heal Psychol 2010 14 43 52 10.1177/1359105308097944 19129336

[B36] Sawatzky R Ratner PA Johnson JL Kopec JA Zumbo BD Self-reported physical and mental health status and quality of life in adolescents: a latent variable mediation model Health and Quality of Life Outcomes 2010 8 17 10.1186/1477-7525-8-17 20128913 PMC2829530

[B37] Lim J Yi J The effects of religiosity, spirituality, and social support on quality of life: a comparison between Korean American and Korean breast and gynecologic cancer survivors Oncology Nursing Forum 2009 36 699 708 10.1188/09.ONF.699-708 19887358

[B38] Helgeson VS Social support and quality of life Quality of Life Research 2003 12 Suppl 1 25 31 12803308 10.1023/a:1023509117524

[B39] Motl RW McAuley E Physical activity, disability, and quality of life in older adults Physical Medicine and Rehabilitation Clinics of North America 2010 21 299 308 10.1016/j.pmr.2009.12.006 20494278

